# Supplementary material for: Biotechnological Test of Plant Growth-Promoting Bacteria Strains for Synthesis of Valorized Wastewater as Biofertilizer for Silvicultural Production of Holm Oak (Quercus ilex L.)
Source: Plants (Basel). 2025 Aug 26;14(17):2654. doi: 10.3390/plants14172654 (PMC12430512; doi:10.3390/plants14172654)
Supplement: Supplementary file 1 [file plants-14-02654-s001.zip › plants-3808519-supplementary.pdf]

Table S1: Complete Sequence QC Results (QIIME2 DADA2).

sample-id	input	filtered	percentage of input passed filter	denoised	merged	percentage of input merged	non-chimeric	percentage of input non-chimeric
**#q2:types**	numeric	numeric	numeric	numeric	numeric	numeric	numeric	numeric
**AM01**	76525	64269	83.98	50798	28401	37.11	21055	27.51
**AM02**	62086	50627	81.54	38412	18915	30.47	13384	21.56
**AM03**	55533	45265	81.51	36749	17408	31.35	13819	24.88
**AM04**	55550	44795	80.64	34734	17505	31.51	12395	22.31
**AM05**	87022	72796	83.65	56079	31781	36.52	20250	23.27
**AM06**	79955	69300	86.67	59122	31872	39.86	21541	26.94
**AM07**	70965	58738	82.77	49282	27133	38.23	19579	27.59
**AM08**	108335	91488	84.45	77992	45943	42.41	27005	24.93
**AM09**	53695	45163	84.11	36065	18773	34.96	13579	25.29

Table S2. Results of PERMANOVA (Adonis) and PERMDISP. P values < 0.1 are interpreted as trends; p values < 0.05 are considered statistically significant.

Factor	Method	Statistic	Df	n	R2	p-value	Permutations
Fertilizer	PERMANOVA	F.Model = 1.393	2	9	0.306	0.060	999
Bacteria	PERMANOVA	F.Model = 1.157	2	9	0.254	0.233	999
Fertilizer	PERMDISP	F = 1.864	2	9	—	0.064	999
Bacteria	PERMDISP	F = 0.424	2	9	—	0.422	999

Table S3. Functions predicted with greater importance in the Random Forest model according to the treatment applied. The KO genes identified as most relevant for the classification of treatments by the Random Forest algorithm are shown, along with their functional function annotated in KEGG.

Treatment	KO	Predicted function (KEGG)
WATER	K07696	two-component system, NarL family, response regulator NreC
	K14988	two-component system, NarL family, secretion system sensor histidine kinase SalK
	K11923	MerR family transcriptional regulator, copper efflux regulator
	K08178	Transportador MFS, familia SHS, transportador de lactato
	K02297	cytochrome o ubiquinol oxidase subunit II [EC:7.1.1.3]
	K00978	glucose-1-phosphate cytidylyltransferase [EC:2.7.7.33]
EDAR	K11941	glucans biosynthesis protein C [EC:2.1.-.-]
EDARST	K06902	MFS transporter, UMF1 family
	K10194	oligogalacturonide transport system permease protein

K18926	MFS transporter, DHA2 family, lincomycin resistance protein
K13318	dTDP-4-ceto-6-desoxi-L-hexosa 4-reductasa [EC:1.1.1.-]
K06971	uncharacterized protein
K06873	uncharacterized protein
K03688	ubiquinone biosynthesis protein
K00672	formylmethanofuran--tetrahydromethanopterin N-formyltransferase [EC:2.3.1.101]

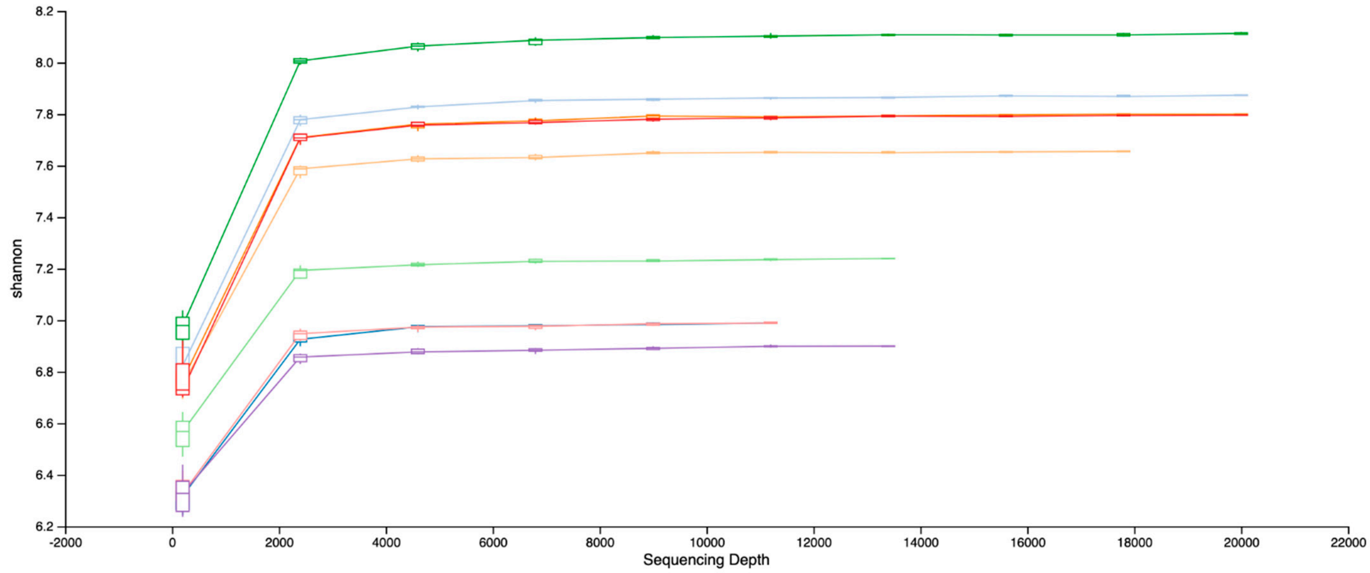

Figure S1: Shannon diversity index rarefaction curves for all samples included in the study. Each line represents an independent sample, showing the variation of the Shannon index as a function of sequencing depth (number of rarefied reads). The curves show that alpha diversity stabilizes before the selected trimming threshold (20,000 readings), confirming that rarefaction does not compromise the diversity estimate. The values represent the average of 10 iterations per depth point.

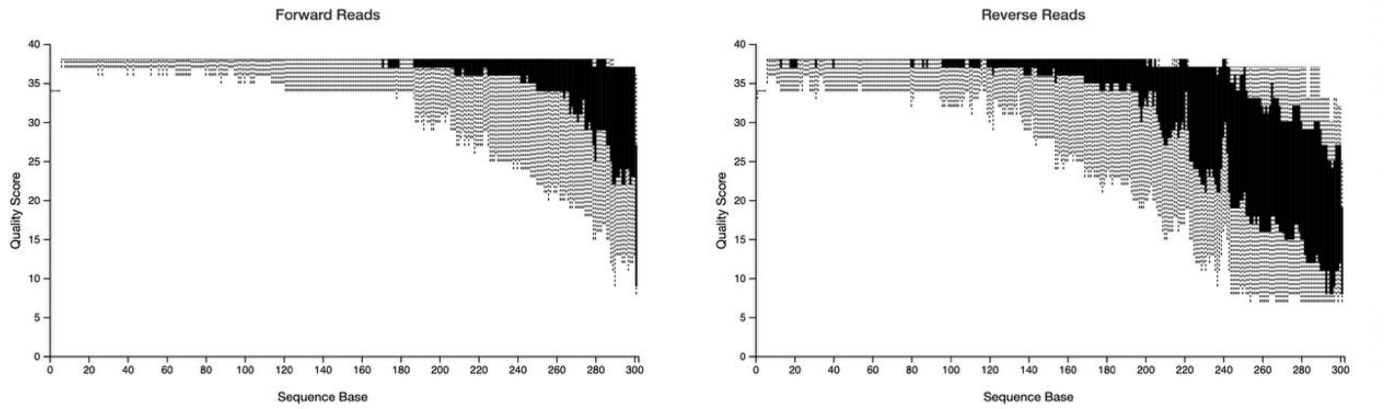

Figure S2. Distribution of quality scores from raw readings.

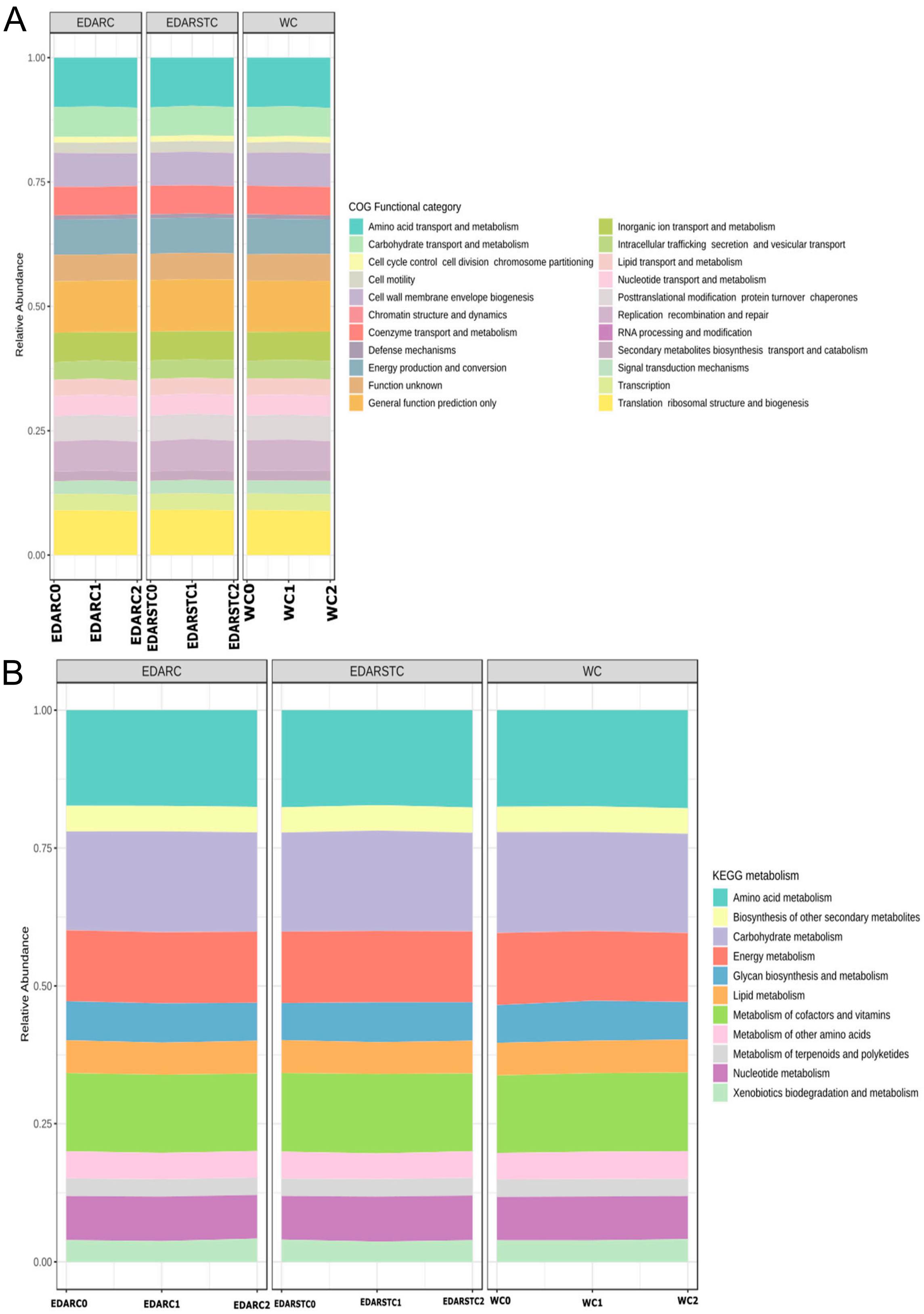

Figure S3: Functional prediction of microbial communities using PICRUSt2. **(A)** Relative abundance of functional categories according to the COG classification. **(B)** Functional distribution of metabolic pathways grouped according to KEGG classification at the category level.

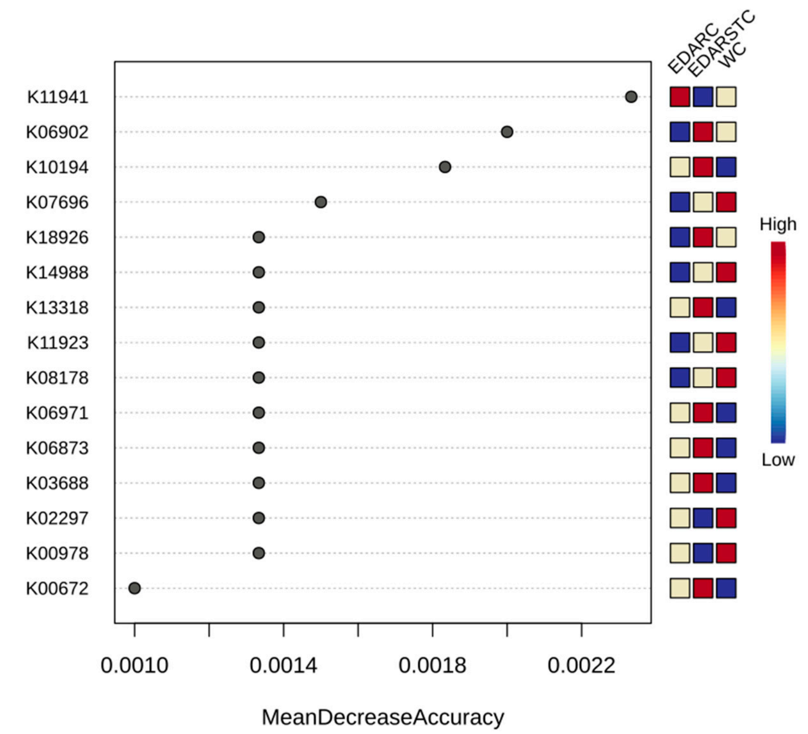

Figure S4. Genes predicted with PICRUSt2 and classified by Random Forest. The x-axis shows the importance of each gene function (KO) in the model, expressed as a mean decrease in classification accuracy (Mean Decrease Accuracy). On the right is a heat map with the standardized relative abundances of the most relevant genes in the different treatments (EDARD, EDARST and WATER) and conditions with or without bacterial strain (C0, C1, C2). The colors represent low (blue) to high (red) values.

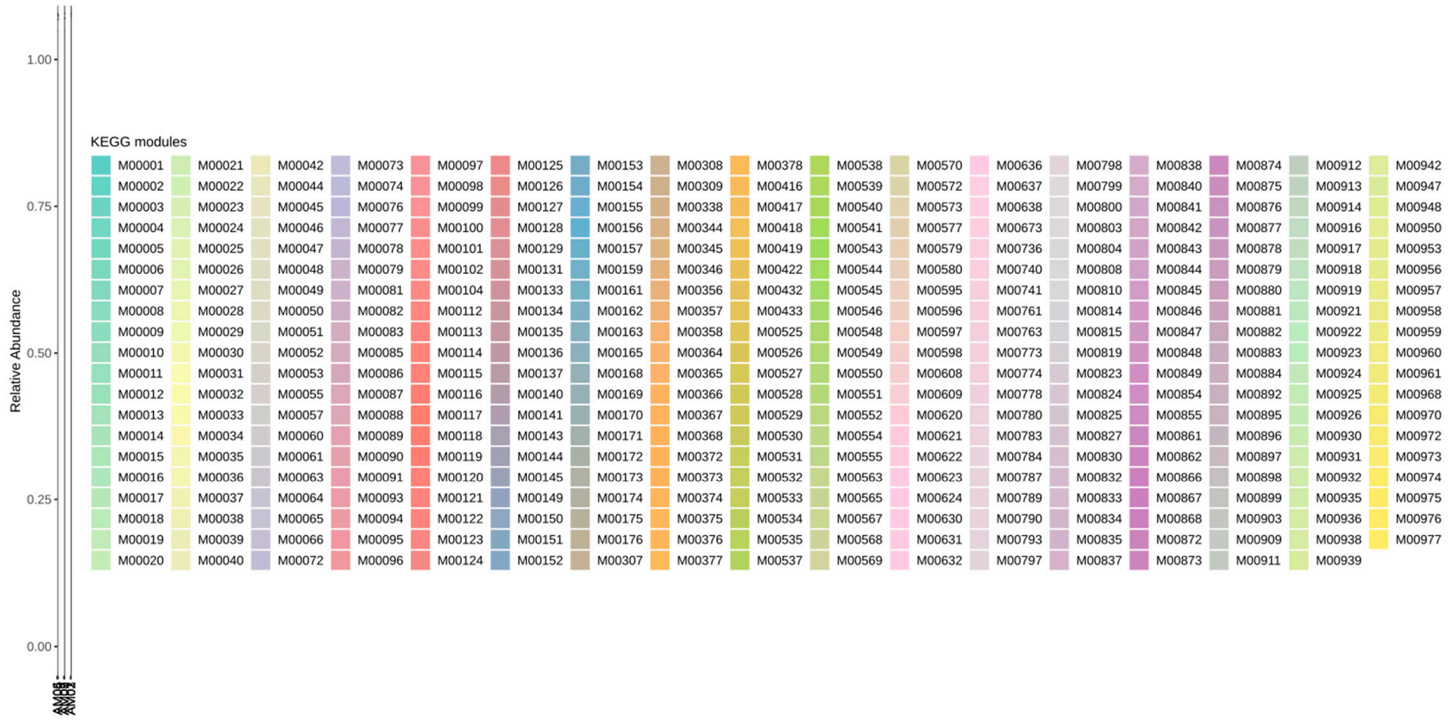

Figure S5. Functional distribution of KEGG modules predicted by PICRUSt2. Relative abundance representation for complete metabolic modules. Homogeneous profile with slight differences between treatments.

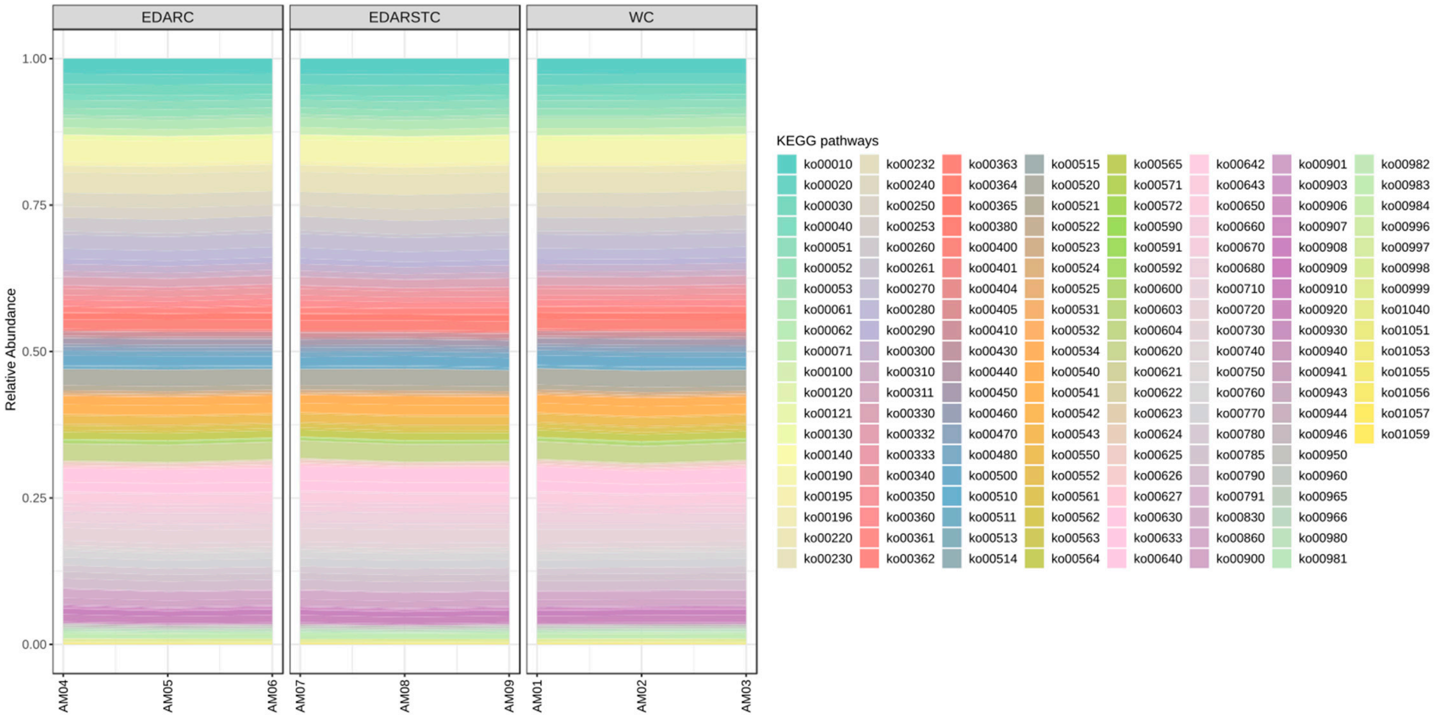

Figure S6. Functional prediction of complete KEGG pathways. Visualization of more than 100 functional pathway-level paths. Most of them have low relative variability between treatments.
